# Regulation of mRNA splicing by MeCP2 via epigenetic modifications in the brain

**DOI:** 10.1038/srep42790

**Published:** 2017-02-17

**Authors:** Tian-Lin Cheng, Jingqi Chen, Huida Wan, Bin Tang, Weidong Tian, Lujian Liao, Zilong Qiu

**Affiliations:** 1 Institute of Neuroscience, Key Laboratory of Primate Neurobiology, State Key Laboratory of Neuroscience, CAS Center for Excellence in Brain Science and Intelligence Technology, Shanghai Institutes for Biological Sciences, Chinese Academy of Sciences, 320 Yue-Yang Road, Shanghai, 200031, China; 2 Department of Biostatistics and Computational Biology, School of Life Science, Fudan University, Shanghai, 200436, China; 3 Shanghai Key Laboratory of Regulatory Biology, School of Life Sciences, East China Normal University, 500 Dongchuan Road, Shanghai, 200241, China

## Abstract

Mutations of X-linked gene Methyl CpG binding protein 2 (*MECP2*) are the major causes of Rett syndrome (RTT), a severe neurodevelopmental disorder. Duplications of *MECP2*-containing genomic segments lead to severe autistic symptoms in human. *MECP2*-coding protein methyl-CpG-binding protein 2 (MeCP2) is involved in transcription regulation, microRNA processing and mRNA splicing. However, molecular mechanisms underlying the involvement of MeCP2 in mRNA splicing in neurons remain largely elusive. In this work we found that the majority of MeCP2-associated proteins are involved in mRNA splicing using mass spectrometry analysis with multiple samples from *Mecp2*-null rat brain, mouse primary neuron and human cell lines. We further showed that *Mecp2* knockdown in cultured cortical neurons led to widespread alternations of mRNA alternative splicing. Analysis of ChIP-seq datasets indicated that MeCP2-regulated exons display specific epigenetic signatures, with DNA modification 5-hydroxymethylcytosine (5hmC) and histone modification H3K4me3 are enriched in down-regulated exons, while the H3K36me3 signature is enriched in exons up-regulated in *Mecp2*-knockdown neurons comparing to un-affected neurons. Functional analysis reveals that genes containing MeCP2-regulated exons are mainly involved in synaptic functions and mRNA splicing. These results suggested that MeCP2 regulated mRNA splicing through interacting with 5hmC and epigenetic changes in histone markers, and provide functional insights of MeCP2-mediated mRNA splicing in the nervous system.

Mutations in the X-linked gene *MECP2*, which encodes methyl-binding protein 2 (MeCP2), are the major causes of neurodevelopmental disorder Rett syndrome (RTT) occurred mainly in girls^1^. RTT patients show severe neurological phenotypes such as mental retardation, functional loss in language, autistic features and motor defects^2^. Several mouse models with *Mecp2* depletion have been established and used as powerful tools to explore the relationship between MeCP2 and Rett syndrome, as *Mecp2*-null mice display phenotypes similar to RTT patients^3^,^4^. Previous studies have shown that pathogenesis of RTT mainly attributed to MeCP2 dysfunctions in the nervous system, which highlights the essential role of MeCP2 for neuronal development and functions^5^,^6^,^7^,^8^. Recently, plenty of studies have investigated the critical roles of MeCP2 in different types of neurons and different cell types of the brain^6^,^9^,^10^,^11^,^12^,^13^; however, the molecular mechanisms for MeCP2 to exert its functions in different cell types remain largely unknown.

MeCP2 was initially identified as a protein showing high affinity to methylated CpG islands^14^,^15^ and further studies revealed that it served as a transcriptional repressor by binding to highly methylated genomic regions^16^. It was also shown that MeCP2 could interact with several transcriptional repressor complexes such as HDAC-mSin3A and NCoR-SMRT complex to repress gene transcription^16^,^17^,^18^. Although it has been confirmed that MeCP2 could repress gene expression in neurons by many studies^19^, studies in *Mecp2*-null mice showed that about 70% genes were down-regulated and this may be due to MeCP2 interaction with CREB to stimulate gene expression^20^. What’s more, it was revealed that MeCP2 could bind to DNA regions containing high level of 5-hydroxymethylcytosine (5 hmC) to promote gene expression^21^. Therefore MeCP2 was considered as a transcriptional modulator to regulate gene expression bi-directionally. In addition, studies also showed that MeCP2 was involved in the regulation of microRNA expression^10^,^22^,^23^, and MeCP2 could interact with Drosha/DGCR8 complex and other factors to modulate microRNA processing^24^,^25^. Subsequent studies demonstrated that MeCP2 could also interact with splicing factors such as Y-box-binding protein 1 (YB-1) to regulate RNA splicing process^26^,^27^. And aberrant RNA splicing events were detected in *Mecp2*^308/Y^ mice that express a truncated MeCP2 protein and recapitulate multiple clinical features of RTT patients^26^. Recent studies in non-neuronal cell lines IMR90 and HCT116 confirmed the involvement of MeCP2 in regulating alternative splicing, by showing that MeCP2 was enriched in included alternative splicing exons (ASEs) with highly methylated status, and MeCP2 knockdown led to dysregulated alternative splicing in these cell lines^28^.

Alternative splicing is a critical post-transcriptional process, which produces divergent mRNA isoforms from a single primary transcript to generate proteins with distinct functions or to modulate protein expression levels via the nonsense-mediated mRNA decay pathway (NMD)^29^,^30^. High-throughput studies showed that alternative splicing process was particularly more common and conserved in the central nervous system as compared to other tissues, and the precise regulation of alternative splicing was critical for divergent aspects of neuronal development via modulation of protein diversity and abundance^31^,^32^,^33^,^34^,^35^.

It was well-established that splicing factors were mainly RNA-binding proteins which regulated alternative splicing at RNA level^36^. Mutations in RNA splicing factors and aberrant alternative splicing have been implicated in various neurological diseases such as amyotrophic lateral sclerosis (ALS), autistic spectrum disorders (ASD) and Rett syndrome^26^,^35^,^37^,^38^,^39^,^40^. Recently, accumulated evidences indicated that chromatin structures and epigenetic modifications such as DNA methylation, hydroxymethylation and histone modifications were also highly involved in mRNA splicing^41^,^42^,^43^,^44^,^45^,^46^. However, it is still unclear how modifications at DNA and chromatin level lead to splicing changes at mRNA level.

In previous studies, MeCP2 was linked to alternative splicing by both interacting with splicing factors and binding to alternative splicing exons with high methylated status. Here we examined the MeCP2-interacting proteins in 293 T cells, cultured mouse cortical neurons, cortical tissues of *Mecp2* null and wild type rat by mass spectrometry and found that the majority were proteins involved in RNA splicing. Furthermore, we analyzed the impact of *Mecp2* knockdown on alternative splicing processes in cultured mouse cortical neurons by RNA sequencing (RNA-Seq) analysis and identified widespread alternative splicing changes upon *Mecp2* deletion. Protein binding status such as MeCP2 binding and RNA polymerase II (Pol II) binding sites, and epigenetic signatures such as 5-methylcytosine (5 mC), 5 hmC and histone modifications in these MeCP2-regulated exons were examined through ChIP-Seq data analysis. It was shown that MeCP2 binding was significantly correlated with Pol II distribution and epigenetic markers including 5 hmC, H3K4me3 and H3K36me3 display specific signatures in MeCP2-regulated exons. Furthermore, functional analysis of genes containing these MeCP2-regulated exons showed that MeCP2-mediated alternative splicing are important for neuronal functions such as synaptic organization, intracellular transport and gene expression regulation and RNA processing. Taken together, These results suggested that in the central nervous system, MeCP2 may serve as a scaffold protein for epigenetic modifications and splicing factors to regulate alternative splicing, and such regulation was critical for normal neuronal functions.

## Results

### MeCP2-associated proteins mainly involved in RNA splicing process

**Figure 1:**
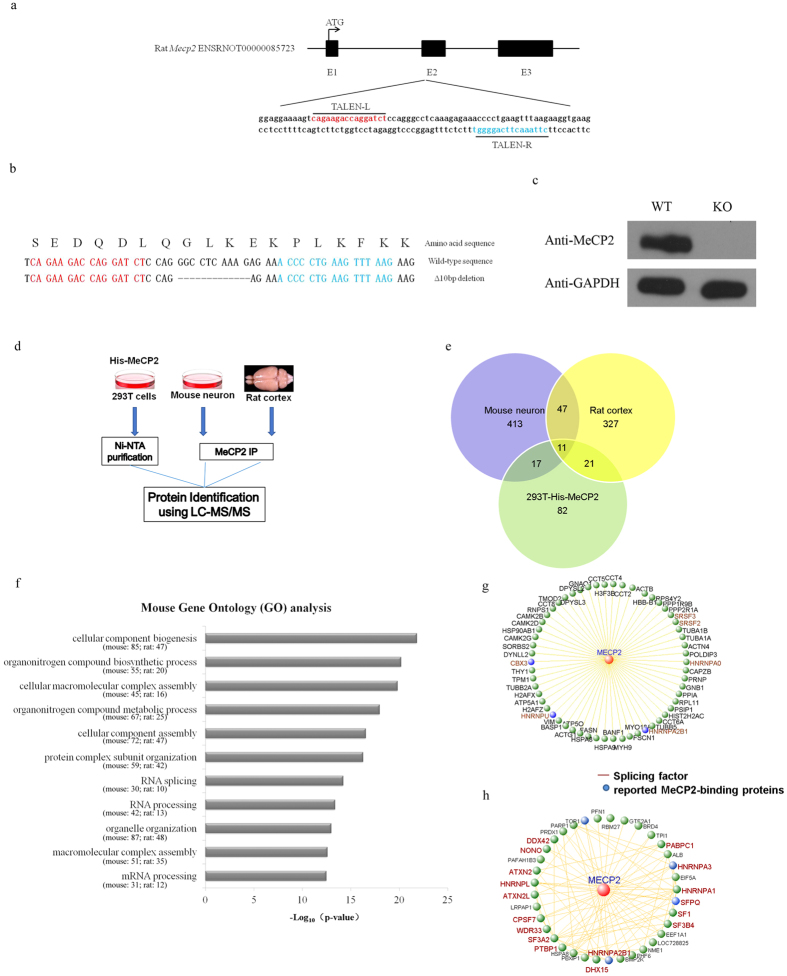
Identification of MeCP2-associated proteins by mass spectrometry analysis and functional annotations. (**a**) Rat *Mecp2* genome locus and TALEN-targeting region design. (**b**) Sequence of the *Mecp2* locus targeted by TALEN-based gene targeting from WT and *Mecp2*-null rat. Corresponding amino acid sequence was annotated. (**c**) MeCP2 expression in *Mecp2*-null rat cortex. Total protein lysates were extracted from *Mecp2*-null rat cortex and related WT littermate cortex and then analyzed by western blot. (**d**) Experimental workflow for immunoprecipitation and mass spectrometry analysis of MeCP2-associated proteins in 293 T cells, mouse cortical neurons and rat cortex. (**e**) Number of MeCP2-associated proteins identified by mass spectrometry in 293 T cells, mouse cortical neurons and rat cortex. (**f**) Mouse GO analysis for MeCP2-associated proteins identified in both cultured mouse cortical neurons and rat cortex. (**g**) Protein-protein interaction network analysis for 58 MeCP2-binding proteins identified in both cultured mouse cortical neurons and rat cortex. (**h**) Protein-protein interaction network analysis for 37 MeCP2-binding proteins identified in 293 T cells which were also detected in either mouse cortical neurons or in rat cortical tissues.

*Mecp2*-null rat was generated using TALEN-based gene targeting technology (Fig. 1a). *Mecp2* disruption with 10 bp deletion was confirmed by PCR and sanger sequencing (Fig. 1b). Furthermore, depletion of MeCP2 protein was confirmed by Western blot using protein lysates collected from *Mecp2*-null (KO) and wild-type (WT) littermate rats (Fig. 1c). To identify MeCP2-associated proteins *in vivo*, we took advantage of the tandem Histidine residues within the MeCP2 protein (a.a. 366–372), suggesting that Ni-NTA resin could be directly used for purifying MeCP2 and its associated proteins from cortical lysates of *Mecp2*-null (KO) and wild-type (WT) littermate rats. We thus performed immunoprecipitation using Ni-NTA resin in protein lysates from *Mecp2*-null and WT rat cortex. Candidate MeCP2-associated proteins found in WT, but not KO lysates, were identified with mass spectrometry analysis (Fig. 1d,e).

To identify MeCP2-binding proteins more accurately, we further performed Ni-NTA purification in 293 T cells expressing His-MeCP2 to identify proteins showing tightly binding affinity to MeCP2. Moreover, we performed immunoprecipitation with anti-MeCP2 antibody in mouse cortical neurons in order to purify endogenous MeCP2-associated proteins in neurons (Fig. 1d). Overall, we identified 131 proteins in 293 T cells while 488 proteins in mouse cortical neurons and 406 proteins in rat cortex, showing high or low affinities to MeCP2 (Fig. 1e). We then performed GO analysis for proteins identified in either mouse cortical neurons or rat cortex, and found that these proteins were mainly enriched in posttranscriptional regulation such as RNA splicing and mRNA processing (Fig. 1f). We further performed protein-protein interaction network analysis for these 58 MeCP2-binding proteins identified in both mouse cortical neurons and rat cortex and revealed that these proteins were tightly connected with splicing factors as the core node (Fig. 1g). Overlap analysis among MeCP2-associated proteins identified in these three experiments showed that 37 MeCP2-binding proteins identified in 293 T cells (excluding Keratin and ribosomal proteins) were also observed in either mouse cortical neurons or in rat cortex and were considered as high-confidence MeCP2-binding proteins. In consistent with above GO analysis, 17/37 high-confidence proteins were splicing factors, indicating that MeCP2 was an important regulator of RNA splicing. Then protein-protein interaction network analysis was performed for these 37 proteins and it was shown that these proteins were closely associated, suggesting that their functions were tightly connected (Fig. 1h).

### Widespread exon usage changes in neurons with MeCP2 knockdown

**Figure 2:**
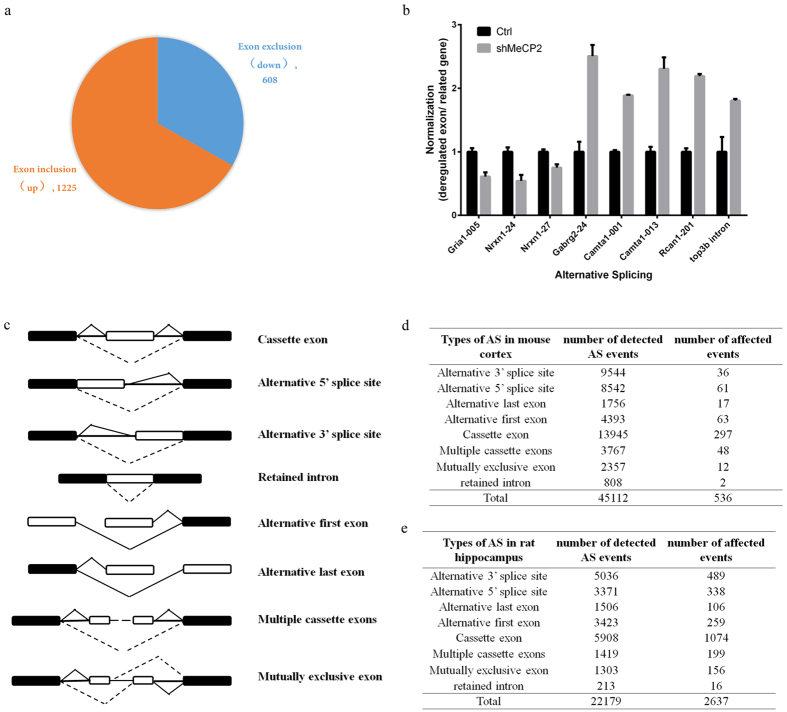
Alternative splicing changes in MeCP2-knockdown neurons. (**a**) Pie chart of exon usage changes in MeCP2-knockdown neurons examined by RNA-Seq and DEXSeq package. (**b**) Real-time PCR analysis of alternative splicing changes in MeCP2-knockdown neurons. Eight exons were chosen based on DEXSeq analysis. Exon-specific primers were designed to determine the expression level of selected exons, and gene-specific primers were used as internal control (**P < 0.01; ***P < 0.001). (**c**) Eight modes of alternative splicing events examined using ASD software. Black boxes representing constitutive exons while white boxes representing alternative spliced exons/regions. Solid lines representing exon inclusion while dotted lines representing exon exclusion. (**d**) Summay of alternative splicing analysis and changed alternative splicing events by ASD software in mouse *Mecp2*-knockdown neurons. (**e**) Summay of alternative splicing analysis and changed alternative splicing events by ASD software in *Mecp2*-null rat hippocampus.

Mass spectra and protein interaction studies revealed that MeCP2-binding proteins were primarily enriched in RNA splicing/processing functions, so we examined the impact of *Mecp2* knockdown on alternative splicing process in mouse cortical neurons by RNA-seq. Mouse cortical neurons were cultured *in vitro* and infected at DIV2 with lentivirus expressing shRNA targeting either mouse *Mecp2* or scrambled sequence. *Mecp2* knockdown efficiency in these neurons was confirmed by both real-time PCR and western blot (Supplementary Fig. 1a,b). RNA deep sequencing was then performed and DEXseq software package was used to analyze exon usage differences between *Mecp2*-knockdown neurons and control neurons. Our analysis identified 1225 exons up-regulated in *Mecp2*-knockdown neurons and 608 exons down-regulated in *Mecp2*-knockdown neurons (P_adjust_ < 0.05) (Fig. 2a). We further analyzed transcriptional levels of genes contained down-regulated or up-regulated exons. It was shown that the expression of 231/482 genes containing down-regulated exons was deregulated (103 were downregulated and 128 were upregulated) while the expression of 477/912 genes containing up-regulated exons was deregulated (185 were downregulated and 292 were upregulated) in *Mecp2*-knockdown neurons. We then selected 8 identified exons whose expression was either increased or decreased in *Mecp2*-knockdown neurons for real-time PCR verification. And our real-time PCR results confirmed the reliability of DEXseq analysis (Fig. 2b). In addition to RNA splicing analysis in *Mecp2*-knockdown neurons, we further analyzed RNA splicing changes in *Mecp2*-null rat hippocampus and observed 1130 exon usage changes as compared to wild-type rat hippocampus (P < 0.01, Supplementary Table 1). In addition to exon usage analysis, we further performed RNA splicing analysis using software ASD described previously^47^ to classify different modes of alternative splicing events. Currently eight modes of alternative splicing events were defined including cassette exon, alternative 5′ splice site, alternative 3′ splice site, retained intron, alternative first exon, alternative last exon, multiple cassette exons and mutually exclusive exon (Fig. 2c). With ASD software, a total of 45112 splicing events were identified and 536 events showed significantly changes in mouse *Mecp2*-knockdown neurons (Supplementary Table 2, Fig. 2d) while in *Mecp2*-null rat hippocampus, a total number of 22179 alternative splicing events were detected and 2637 events changed significantly as compared to wild-type rat hippocampus (Supplementary Table 3, Fig. 2e).

We further compared the exons identified by DEXseq with exons identified by ASD software. It was shown that 32 differentially expressed exons revealed by both softwares and at least 17 exons were located in genes critical for neuronal development and synaptic functions (Fig. S1c).

### Distribution of MeCP2 and Pol II proteins in MeCP2-regulated exons

**Figure 3:**
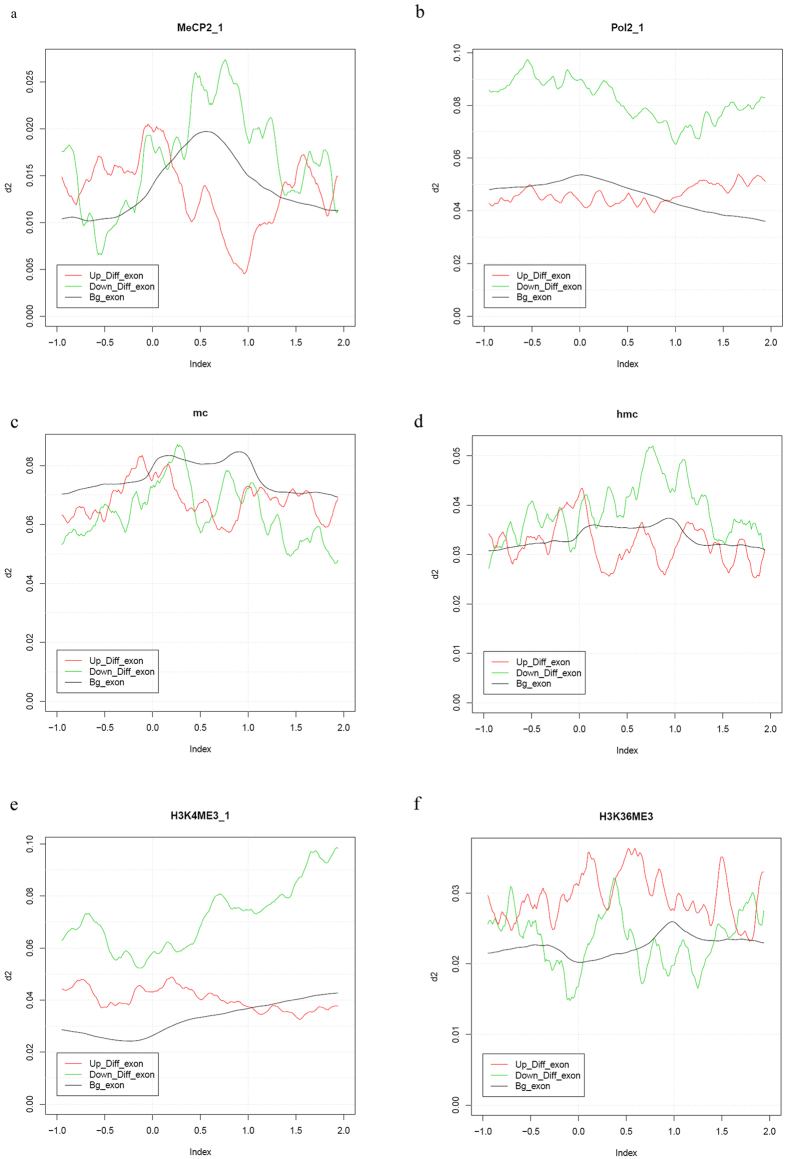
Protein distribution and epigenetic signatures in MeCP2-regulated exons. (**a**) Average diagram of MeCP2 ChIP-Seq data in exons which were up-regulated, down-regulated or unchanged in MeCP2-knockdown neurons. The Y axis represents the degree of ChIP-Seq data relative to input, and the X axis indicates the relative distance of exons, in which each exon was equally divided into 100 parts, with 0.0 representing the start position of exon and 1.0 represents the end position of exon. (**b**) Average diagram of Pol II ChIP-Seq data in exons which were up-regulated, down-regulated or unchanged in MeCP2-knockdown neurons. (**c**) Average diagram of 5 mC status in exons which were up-regulated, down-regulated or unchanged in MeCP2-knockdown neurons. (**d**) Average diagram of 5 hmC status in exons which were up-regulated, down-regulated or unchanged in MeCP2-knockdown neurons. (**e**) Average diagram of H3K4me3 ChIP-Seq data in exons which were up-regulated, down-regulated or unchanged in MeCP2-knockdown neurons. (**f**) Average diagram of H3K36me3 ChIP-Seq data in exons which were up-regulated, down-regulated or unchanged in MeCP2-knockdown neurons.

We further examined the distribution of MeCP2 and RNA polymerase II (Pol II) proteins in MeCP2-regulated exons identified in *Mecp2*-knockdown neurons. ChIP-seq data for MeCP2 and Pol II binding status in neurons under similar conditions was selected and re-analyzed for up-regulated, down-regulated and unchanged exons of MeCP2-knockdown neurons respectively. Our analysis revealed that both MeCP2 and Pol II were highly enriched in down-regulated exons (Fig. 3a,b, Supplementary Fig. 2a,b), indicating that MeCP2 and Pol II were critical for exon inclusion in normal conditions. Intriguingly, though the distribution of Pol II protein in up-regulated exons was similar to unchanged exons, the distribution of MeCP2 was significantly higher in splice acceptor site of up-regulated exons as compared to unchanged exons (Fig. 3a,b, Supplementary Fig. 2b), which suggested that MeCP2 may modulate exon exclusion in normal conditions via mechanisms independent of Pol II-mediated transcriptional elongation.

### Distribution of epigenetic markers in MeCP2-regulated exons

MeCP2 was a methyl/hydroxymethyl-DNA binding protein and closely associated with nucleosome. As DNA methylation status and histone modifications were involved in alternative splicing process, we further examined whether epigenetic markers such as 5 mC, 5 hmC and histone methylation such as H3K4me3 and H3K36me3 distributed differentially in MeCP2-regulated exons. ChIP-seq data for 5 mC, 5 hmC, H3K4me3 and H3K36me3 in similar conditions as ours was described previously and chosen to determine their distribution in up-regulated, down-regulated and unchanged exons of MeCP2-knockdown neurons. It was shown that DNA methylation level in either down-regulated or up-regulated exons were lower than that in unchanged exons (Fig. 3c). However, DNA hydroxymethylation level was significantly higher in down-regulated exons as compared to up-regulated or unchanged exons (Fig. 3d). These results indicated that MeCP2 binding to down-regulated exons in neurons might be dependent on DNA hydroxymethylation but not DNA methylation.

In addition to DNA methylation status, distribution of histone modifications such as H3K4me3 and H3K36me3 were also examined and it was shown that H3K4me3 was highly enriched in down-regulated exons while H3K36me3 was enriched in up-regulated exons (Fig. 3e,f). It has been reported previously that H3K4me3 was involved in exon inclusion while H3K36me3 was involved in exon exclusion^48^, so our results suggested that MeCP2 might be critical for the H3K4me3- and H3K36me3-mediated alternative splicing.

### Functional analysis of genes containing MeCP2-regulated exons

**Figure 4:**
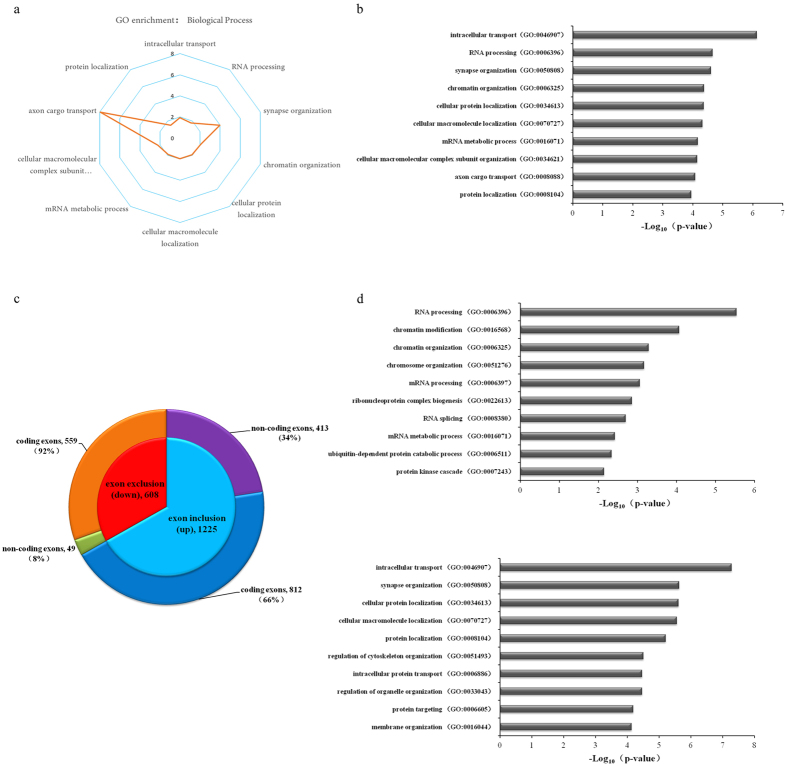
Functional annotations for genes containing MeCP2-regulated exons. (**a**) Radar chart for GO analysis of the genes containing MeCP2-regulated exons identified in mouse cortical neurons. (**b**) GO analysis for the genes containing MeCP2-regulated exons. (**c**) Pie chart of the exon classifications for MeCP2-regulated exons based on the exon’s coding ability. (**d**) GO analysis for the genes containing MeCP2-regulated coding exons and for the genes containing MeCP2-regulated non-coding exons, respectively.

It has been reported that *loss-of-function* of MeCP2 in cultured neurons or mouse models impaired neuronal functions in different aspects including dendritic growth, axon transport and synaptic functions^6^,^49^,^50^. Here we further performed functional enrichment analysis to investigate the functional importance of genes containing MeCP2-regulated exons in neurons. It was shown that genes containing MeCP2-regulated exons were highly enriched in axon cargo transport and synaptic organization (Fig. 4a,b). In addition, GO analysis was also performed for genes containing MeCP2-regulated exons identified in rat cortex and revealed that axon cargo transport was also the highly enriched GO terms (Fig. S3a). We further compared genes containing MeCP2-regulated exons to curated database of genes encoding synaptic proteins or candidate gene lists of autism spectrum disorders. Significant overlap was observed with genes encoding synaptic proteins including synaptome, postsynaptic proteome, presynaptic proteome and several complexes such as NMDAR and mGluR5 (Supplementary Table 4). Overlapping with candidate gene sets of autism spectrum disorders (SFARI Autism database) was also significant (>10%), in consistent with the implication of MeCP2 in autism spectrum disorders (Supplementary Table 4). In addition, significant functional enrichment was also observed in intracellular transport, RNA processing, chromatin organization (Fig. 4b). These results suggested that MeCP2-mediated alternative splicing was important for normal neuronal functions and likely involved in epigenetic or post-transcriptionally regulation.

Exons were further annotated according to protein-coding ability of related transcripts and divided into coding exons which was in the transcripts of protein-coding mRNAs or non-coding exons only in non-coding transcripts including non-mediated decay or processed transcripts. It was speculated that changes of coding exons usage may influence protein diversity while non-coding exons might be involved in the regulation of translation and protein expression, so MeCP2-regulated exons were further classified into these two classes for functional analysis. It was shown that exons down-regulated in MeCP2-knockdown neurons were mainly coding exons (559/608) while a large fraction of non-coding exons were up-regulated (413/462) (Fig. 4c). GO functional analysis showed that non-coding exons were mainly enriched in functions associated with chromatin and RNA regulation such as RNA processing, chromatin modification and chromatin organization; while coding exons were enriched in neuronal physiological functions such as intracellular transport and synapse organization (Fig. 4d). These results indicated that MeCP2-mediated RNA splicing had divergent functional consequences, further confirming the multifaceted functions of MeCP2.

## Discussion

Several studies confirmed the involvement of MeCP2 in regulating alternative splicing and provided insights into the underlying mechanisms, by showing that MeCP2 may modulate alternative splicing through its interaction with both splicing factors and epigenetic markers such as 5 mC^26^,^28^,^51^. However, comprehensive analysis for the role of MeCP2 in alternative splicing in neurons remained elusive. Here we explored the potential mechanisms underlying MeCP2-mediated alternative splicing at protein, RNA and DNA level in neurons using mass spectrum, RNA-Seq and ChIP-Seq data re-analysis. Our results showed that MeCP2 depletion in cultured cortical neurons led to significant splicing changes by RNA-Seq, confirming the critical role of MeCP2 in alternative splicing regulation. Mass spectrum for MeCP2-binding proteins showed that MeCP2 could interact with transcriptional regulators, splicing factors and chromatin-related modifiers (Supplementary Fig. 3), which suggested that MeCP2 might serve as a bridge for chromatin-related modifiers and splicing factors to modulate alternative splicing. Moreover, ChIP-Seq data re-analysis for MeCP2, 5 mC and 5 hmC revealed that MeCP2 distribution in down-regulated exons of *Mecp2*-knockdown neurons was positively correlated with 5 hmC but not 5 mC, which is not in consistent with studies in non-neuronal cell lines IMR90 and HCT116^28^. This inconsistency might reflect that MeCP2 functions varied depending on cell types, and is consistent with previous studies showing that the role of 5 hmC in alternative splicing regulation was tissue specific^46^. We further analyzed ChIP-Seq data for Pol II and confirmed that MeCP2-mediated alternative splicing might be coupled to Pol II-regulated transcriptional elongation^28^. Pol II distribution analysis in this study also confirmed the reliability of our ChIP-data re-analysis. We then further examined the ChIP-Seq data for H3K4me3 and H3K36me3, which has been implicated in the regulation of exon inclusion and exon skipping respectively, and revealed that H3K4me3 was highly enriched in down-regulated exons while H3K36me3 was enriched in up-regulated exons of *Mecp2*-knockdown neurons. As MeCP2 was enriched in down-regulated exons and MeCP2 could interact with CHD1 and PARP1 (Supplementary Fig. 3), which were H3K4me3-related proteins, it is possible that MeCP2 might be involved in H3K4me3-mediated exon inclusion. Taken together, MeCP2 might serve as a scaffold protein for alternative splicing, by “translating” epigenetic modifications into signals to guide splicing factors for splicing regulation.

In addition to investigations about mechanisms underlying MeCP2-mediated alternative splicing, we also examined the functional enrichment of genes containing MeCP2-regulated exons in cultured cortical neurons. It was shown that MeCP2-mediated alternative splicing was highly enriched in two major functional classes: one is associated with neuronal physiological functions such as intracellular transport, axon cargo transport and synaptic organization while another is closely related to chromatin and RNA regulation such as chromatin structure, RNA processing and splicing. We noticed that according to the protein-coding ability, exons could be further divided into coding exons and non-coding exons, and functional enrichment reanalysis for coding and non-coding exons regulated by MeCP2 showed that coding exons mainly enriched in neuronal physiological functions while non-coding exons mainly enriched in chromatin and RNA regulatory functions.

Moreover, we further found that most of the MeCP2-regulated non-coding exons were up-regulated in MeCP2-knockdown neurons (413/462). As non-coding exons might inhibit gene expression via protein translational regulation, it is possible that in normal neurons, MeCP2 inhibited the inclusion of non-coding exons to promote gene expression while *Mecp2* knockdown led to repression of gene expression at protein level. Our analysis thus indicated that MeCP2-mediated alternative splicing might influence neuronal functions via two different strategies: one is to regulate protein diversity by coding exons, and another is to regulate protein expression by non-coding exons. Our studies not only systematically explore the mechanisms underlying MeCP2-mediated alternative splicing, but also provide insights into the roles of MeCP2-mediated alternative splicing, which could influence both protein diversity and protein expression level in neurons.

## Materials and Methods

### Ethical approval and informed consent

The experimental protocol and procedures followed the guidelines and regulations issued by SIBS, CAS and were approved by Shanghai Institutes for Biological Sciences, Chinese Academy of Sciences.

### *Mecp2* knockdown in neurons

The shRNA specifically against mouse *Mecp2* and related lentivirus preparation has been described previously^24^. Briefly, embryonic day 15–16 mouse cortical cells were isolated and cultured in 6-well plates. Lentivirus expressing either shRNA against mouse *Mecp2* or control shRNA was added at day *in vitro* (DIV) 2, and neurons were cultured for another 3–5 days. Total RNAs/proteins were collected for further analysis.

### Generation of *Mecp2*-null rat

*Mecp2*-null rat was generated by transcription activator-like effector nucleases (TALENs)-mediated gene targeting. In brief, TALENs mRNAs specifically targeting against exon2 of the rat *Mecp2* gene were designed, tested and prepared by the Beijing Biocytogen Co. Ltd. Rat zygotes were injected with *Mecp2* TALENs mRNAs and were then implanted into surrogate rat mothers. *Mecp2* locus was determined in F0 rats by PCR and sanger sequencing and rats carrying disrupted *Mecp2* genome locus were chosen for F1 preparation. MeCP2 expression in F1 rats was determined by western blot. *Mecp2*-null rats and wild-type (WT) littermates were used for subsequent experiments.

### Purification of MeCP2 protein complexes

MeCP2 protein complexes were purified as described previously^52^. Briefly, His-MeCP2 expression plasmid was transfected into 293 T cells and purified by Ni-NTA His binding resin (Millipore, Bedford, MA, USA) was used to purify overexpressed His-MeCP2 in 293-T cells, and after washing extensively for four times with Urea buffer, mass spectrometry was performed to identify the proteins closely associated with MeCP2.

### Immunoprecipitation in cultured mouse cortical neurons and in rat cortex

As described previously^52^, cultured mouse cortical neurons were collected and lysed in RIPA buffer and MeCP2 antibody (Cell signaling technology, Beverly, MA, USA) was used for protein immunoprecipitation and normal rabbit IgG (Santa Cruz, CA, USA) was used as negative control.

Immunoprecipitation in rat cortex was performed as following: *Mecp2*-null rat cortex and WT littermate cortex were collected and lysed with RIPA buffer, and were purified by Ni-NTA His binding resin (Millipore, Bedford, MA, USA) due to the tandem Histidine residues in the MeCP2 protein.

### Trypsin digestion

The purified protein was suspended and reduced in 50 μl lysis buffer (7 M urea, 2 M thiourea, 10 mM HEPES, 1 mM sodium orthoranadate, 5 mM sodium fluoride, 5 mM β-glycerophosphate, 10 mM DTT, pH 8.0, then alkylated with 50 mM iodoacetamide. After reducing urea concentration to 2 M, the samples were digested with trypsin (Promega, USA) at 37 °C (1:100 w/w) overnight. The digested peptide mixtures were acidified with trifluoroacetic acid (TFA) at 1% final concentration and desalted by reversed-phase C18 Sep-Pak cartridge (Millipore, USA).

### Mass spectrometry analysis

Peptides were loaded and separated on an EASY-nLC1000 LC (Thermo Scientific) coupled on line to the Q-Exactive Orbitrap mass spectrometer (Thermo Scientific). The microcapillary trap column was constructed in a 25 mm × 75 silica capillary, packed with 5-μm Luna C18 stationary phase (Phenomenex). The analytical column was constructed in a 150 mm × 75 μm silica capillary tip pulled with a column puller and packed with 3-μm Luna C18 stationary phase. The organic gradient was over 120 min using buffers A and B. Buffer A contained 2% acetonitrile with 0.1% acetic acid. Buffer B contained 98% acetonitrile with 0.1% acetic acid. The separation was performed in 90 min at a flow rate of 300 nl/min, with a gradient of 2% to 6% buffer B in 1 min, followed by 6% to 24% in 61 min, 24% to 100% in 14 min, then 100% buffer B for 5 min and 100% to 0% in 1 min and finally 0% buffer B in 8 min. Full MS were acquired at a resolution of 70,000 with an AGC target value of 3 × 106 and a maximum injection time of 100 ms. Target value for the MS/MS scans was 1 × 105 charges with a resolution of 17,500 and a maximum injection time of 50 ms. The raw files were analyzed by MaxQuant software (version 1.5.3.8) against the latest release of the human, mouse or rat Uniprot database respectively (http://www.uniprot.org/proteomes/).

### RNA-Seq

Hippocampal tissue from *Mecp2*-null rats or wild-type rats at postnatal 30 days and neurons infected with lentivirus expressing either shRNA against mouse *Mecp2* or control shRNA were prepared in biological triplicate and total RNA was isolated by TRIzol reagent (Invitrogen). Then RNase-free DNaseI was used to remove potential genomic DNA contamination and polyA + mRNA was isolated using Dynabeads oligo(dT) for cDNA preparation. Superscript II reverse transcriptase (Invitrogen) and random hexamer primers were used for double-stranded cDNA synthesis. Then mRNA-seq libraries for mouse neurons were established according to the standard Illumina protocol. RNA-seq was performed using Illumina Hiseq 2000 > 45 million 2 × 100 reads per sample was produced and alignment was performed using mouse genome database GRCm38 version 67. Raw data for RNA-seq was submitted to NCBI GEO database #79993. Complementary DNA (cDNA) libraries of rat hippocampus for single-end sequencing were prepared using Ion Total RNA-Seq Kit v2.0 (Life Technologies) according to the manufacturer’s instructions. The cDNA libraries were then processed for the Proton Sequencing process according to the commercially available protocols. Samples were diluted and mixed, the mixture was processed on a OneTouch 2 instrument (Life Technologies) and enriched on a OneTouch 2 ES station (Life Technologies) for preparing the template-positive Ion PI™ Ion Sphere™ Particles (Life Technologies) according to Ion PI™ Template OT2 200 Kit v2.0 (Life Technologies). After enrichment, the mixed template-positive Ion PI™ Ion Sphere™ Particles of samples was loaded on to 1 P1v2 Proton Chip (Life Technologies) and sequenced on Proton Sequencers according to Ion PI Sequencing 200 Kit v2.0 (Life Technologies).

### Quantitative Real-Time PCR

Quantitative real-time PCR assay was performed as described previously. Briefly, total RNA was isolated by TRIzol reagent (Invitrogen) and then converted to cDNA using 5XPrimeScript RT Master Mix (TAKARA). SYBR Premix from Toyobo was used with Rotor-Gene Q machine (QIANGEN). Data was analyzed with comparative C_T_ method. Primers used in this study were as follows:

Gria1-005 for: ACATTGAGCAACGCAAGC;

Gria1-005 rev: CCCTGCTCGTTCAGTTTTAAC;

Gria1 for (internal control): GTCCGCCCTGAGAAATCCAG;

Gria1 rev (internal control): CTCGCCCTTGTCGTACCAC;

Nrxn1 for (internal control): CGGAGACCCCAGTCCTATGG;

Nrxn1 rev (internal control): AACATCATCGGATGCAAATGGA;

Nrxn1-024 for: AGGCATTGGACACGCTATG;

Nrxn1-024 rev: TTACTGACTGGTGACCCTGG;

Nrxn1-027 for: TGCCAAAACTGGTCCATGC;

Nrxn1-027 rev: GCAAGTCAGCTTTCATCAATGC;

Gabrg2-027 for: CTTTGGTGGAGTATGGCAC;

Gabrg2-027 rev: AAGGAAAACATCCGAAGAAGAG;

Gabrg2 for (internal control): CAGACTTACATTCCCTGCAC;

Gabrg2 rev (internal control): CTTGGGCAGAGATTTTCTGG;

Camta1 for (internal control): CTATGTCCATTCCTCCATCATAC;

Camta1 rev (internal control): TAGCCCACTCCTTCTTGTC;

Camta1-013 for: CCGAAAAAGCTGCTTGAATGTC;

Camta1-013 rev: AAGGTAATGCAGGTCAGGGG;

Camta1-001 for: AGCGTTTCCCAAAGTGTATTC;

Camta1-001 rev: ACTTGAACATTTCGGCAGAC;

Rcan1-201 for: ACAATTTTAGCTCCCTGATTGC;

Rcan1-201 rev: GCTCTTAAAATACTGGAAGGTGG;

Rcan1 for (internal control): AAACTTCAGCAACCCCTTATC;

Rcan1 rev (internal control): CAGGTGTGAACTTCCTATGTG;

Top3b-intron for: GAAAGGAACACCGTACAGC;

Top3b-intron rev: TCACAACTAGGAATAGGATAGGG;

Top3b for (internal control): GTGCTCATGGTAGCAGAAAAG;

Top3b rev (internal control): GCAAAGGTTCCTGTGTACTTG.

### Exon usage analysis

Alternative spliced exons RNA-Seq reads were aligned to the mouse genome by the company, and bam files were obtained. We used the DEXSeq package^53^ in Bioconductor to identify significantly differentially regulated exons in MeCP2 knockdown neurons based on these bam files, according to Version 67 of the ensemble annotation GTF file for mouse. Exons with a p-value less than or equal to 0.05 after multi-test correction were considered significantly differentially regulated. Significantly downregulated exons were considered as “inclusive” exons, while significantly up-regulated exons were “exclusive” exons.

### Alternative Splicing Detection

We selected the Alternative Splicing Detector (ASD, available on http://www.novelbio.com/asd/ASD.html) as the tool to detect the differentially alternative splicing cases based on the bam file after mapping according to the P-Value threshold (P-Value < 0.05)^47^.

### ChIP-Seq data analysis

ChIP-Seq data in BigWig format for MeCP2 binding sites in cultured embryonic mouse cortical neurons were downloaded from the GEO database (GSE31851^54^). ChIP-Seq data in BigWig format for PolII binding and histone markers in embryonic mouse cortical neurons were downloaded from the GEO database (GSE21161^55^). DNA methylation data (5mc and 5hmc) and ChIP-Seq data for histone markers in BigWig format in embryonic mouse cortex neurons were downloaded from the GEO database (GSE38118^56^). The BigWig files were then transformed to bedgraph format, and narrow peaks were called by using MACS^57^. The generated peak regions for PolII, histone markers and DNA methylation were then mapped to the regions of the exclusive exons and the inclusive exons. Each exon region was extended as the length of the exon region to both the upstream and the downstream of the exon, and were also mapped with the generated peak regions. Results for all the exclusive exons and all the inclusive exons were combined together to draw figured to display the epigenetic dynamics in and around these exons. A sliding window of 10 bp was used to draw each point. The same was applied to all exons used in this analysis as the background. KS test and T test were used to examine whether there was a significant different among the distributions of each histone marker/Methylation type/Pol II binding in exclusive exons, inclusive exons and all background exons used in this analysis.

### Functional enrichment analysis

Genes containing exclusive exons and inclusive exons were obtained. Functional enrichment analysis was applied to these genes by using the online server DAVID (https://david.ncifcrf.gov).

### Exon annotations

Exons were annotated according to ensemble annotation file using DEXSeq. Then these exons were compared with protein-coding exons annotated in ensemble database. Those showing no overlap with protein-coding exons were classified as non-coding exons.

### Comparison between genes containing MeCP2-regulated exons and functional gene list

The overlap of genes containing MeCP2-regulated exons with different functional gene lists was analyzed as described previously^58^. In brief, gene lists of postsynaptic proteome, NMDAR complex, mGluR5 complex, AMPAR complex and synaptome were downloaded from http://www.genes2cognition.org/, the pre-synaptic proteome has been described in Croning *et al*., Table S4^59^. Gene list for autism spectrum disorders was from Simons SFARI Gene database^60^. Statistical analysis for overlap between genes containing MeCP2-regulated exons and different gene lists was determined by Fisher’s exact test.

## Additional Information

**How to cite this article**: Cheng, T.-L. *et al*. Regulation of mRNA splicing by MeCP2 via epigenetic modifications in the brain. *Sci. Rep.* **7**, 42790; doi: 10.1038/srep42790 (2017).

**Publisher's note:** Springer Nature remains neutral with regard to jurisdictional claims in published maps and institutional affiliations.

## Supplementary Material

Supplementary Information

Supplementary Table 1

Supplementary Table 2

Supplementary Table 3
