# Supplementary material for: Regulation of mRNA splicing by MeCP2 via epigenetic modifications in the brain
Source: Sci Rep. 2017 Feb 17;7:42790. doi: 10.1038/srep42790 (PMC5314398; doi:10.1038/srep42790)
Supplement: Supplementary Information [file srep42790-s1.pdf]

Regulation of mRNA splicing by MeCP2 via epigenetic modifications in the brain

Tian-Lin Cheng[1,*], Jingqi Chen[2], Huida Wan[3], Bin Tang[3], Weidong Tian[2], Lujian Liao[3,*], Zilong Qiu[1,*]

[1]Institute of Neuroscience, Key Laboratory of Primate Neurobiology, State Key Laboratory of Neuroscience, CAS Center for Excellence in Brain Science and Intelligence Technology, Shanghai Institutes for Biological Sciences, Chinese Academy of Sciences, 320 Yue-Yang Road, Shanghai, China, 200031

[2]Department of Biostatistics and Computational Biology, School of Life Science, Fudan University, Shanghai, China, 200436

[3]Shanghai Key Laboratory of Regulatory Biology, School of Life Sciences, East China Normal University, 500 Dongchuan Road, Shanghai, China 200241

*Co-corresponding authors: Tian-Lin Cheng (tlcheng@ion.ac.cn) , Lujian Liao (ljliao@bio.ecnu.edu.cn), Zilong Qiu (zqiu@ion.ac.cn)

Supplementary Figure 1.

a-b. MeCP2 expression in cultured mouse cortical neurons infected with lentivirus expressing shRNA targeting either mouse *Mecp2* or scrambled sequence at mRNA (a) and protein level (b).

c. Genes containing exons identified by both ASD and DEXseq software in mouse cortical neurons.

Supplementary Figure 2. Genomic location of MeCP2, Pol II, H3K4me3

A. Average diagram of MeCP2 ChIP-Seq data2 in exons which were upregulated, downregulated or unchanged in MeCP2-knockdown neurons.
B. Average diagram of Pol II ChIP-Seq data2 in exons which were upregulated, downregulated or unchanged in MeCP2-knockdown neurons.
C. Average diagram of H3K4me3 ChIP-Seq data2 in exons which were upregulated, downregulated or unchanged in MeCP2-knockdown neurons.
D. Average diagram of H3K4me3 ChIP-Seq data3 in exons which were upregulated, downregulated or unchanged in MeCP2-knockdown neurons.

Supplementary Figure 3

a. Histogram and radar chart for GO analysis of the genes containing MeCP2-regulated exons identified in rat cortex.

b. Functional annotations of MeCP2-binding proteins identified in 293T cells by Ni-NTA purification strategy.

Supplementary Table 1 RNA splicing changes in *Mecp2*-null rat hippocampus as compared to wild-type rat analyzed by DEXseq software.

Supplementary Table 2 RNA splicing changes in *Mecp2*-knockdown mouse cultured cortex neurons as compared to wild-type neurons analyzed by ASD software.

Supplementary Table 3 RNA splicing changes in *Mecp2*-null rat hippocampus as compared to wild-type rat analyzed by ASD software.

Supplementary Table 4 Overlap between genes containing MeCP2-regulated exons and different gene lists

The numbers in *Gene number* line means protein number in database (number of genes encoding related proteins identified in RNA-seq data)

A

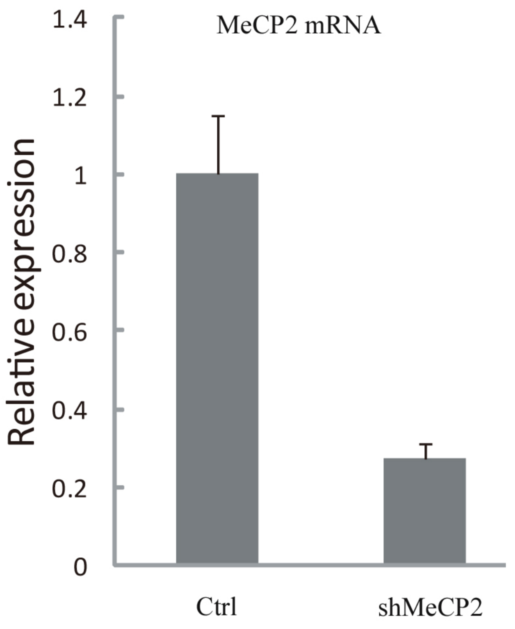

B

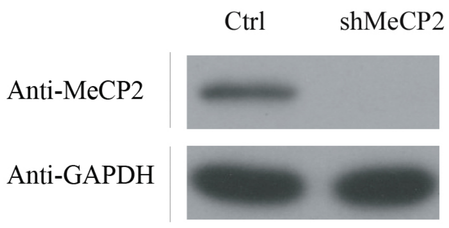

C

Genes containg exons identified by both ASD and DEXseq software	
Celf6	Amn1
St3gal3	Cnnm3
Mff	Enah
Rcan1	Fam49b
Nek2	Gapvd1
Dclk1	Mcf2l
Fyn	Mfsd6
Gabrg2	Osbpl9
Spna2	Ppig
Wdfy3	Ptk2
Runx1t1	Rcbtb2
Pfkfb3	Sirt2
Eps15	St6galnac6
Camta1	Ttll5
Pdzrn3	1810012P15Rik
Rims1	
Syt7	

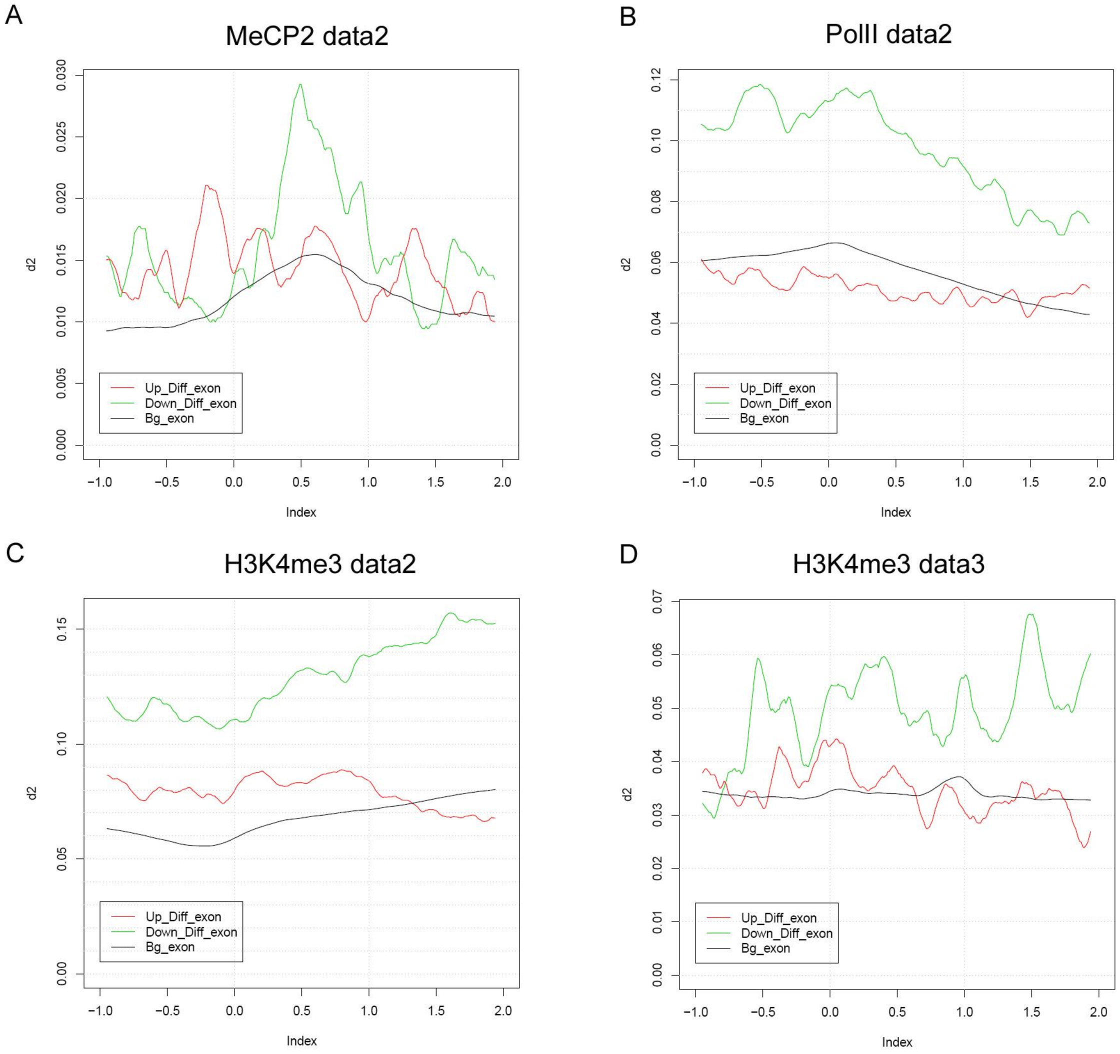
A
MeCP2 data2
0.000
0.005
0.010
0.015
0.020
0.025
0.030
d2
-1.0
-0.5
0.0
0.5
1.0
1.5
2.0
Index
Up_Diff_exon
Down_Diff_exon
Bg_exon
B
PolII data2
0.00
0.02
0.04
0.06
0.08
0.10
0.12
d2
Index
Up_Diff_exon
Down_Diff_exon
Bg_exon
C
H3K4me3 data2
0.00
0.05
0.10
0.15
d2
Index
Up_Diff_exon
Down_Diff_exon
Bg_exon
D
H3K4me3 data3
0.00
0.01
0.02
0.03
0.04
0.05
0.06
0.07
d2
Index
Up_Diff_exon
Down_Diff_exon
Bg_exon

A

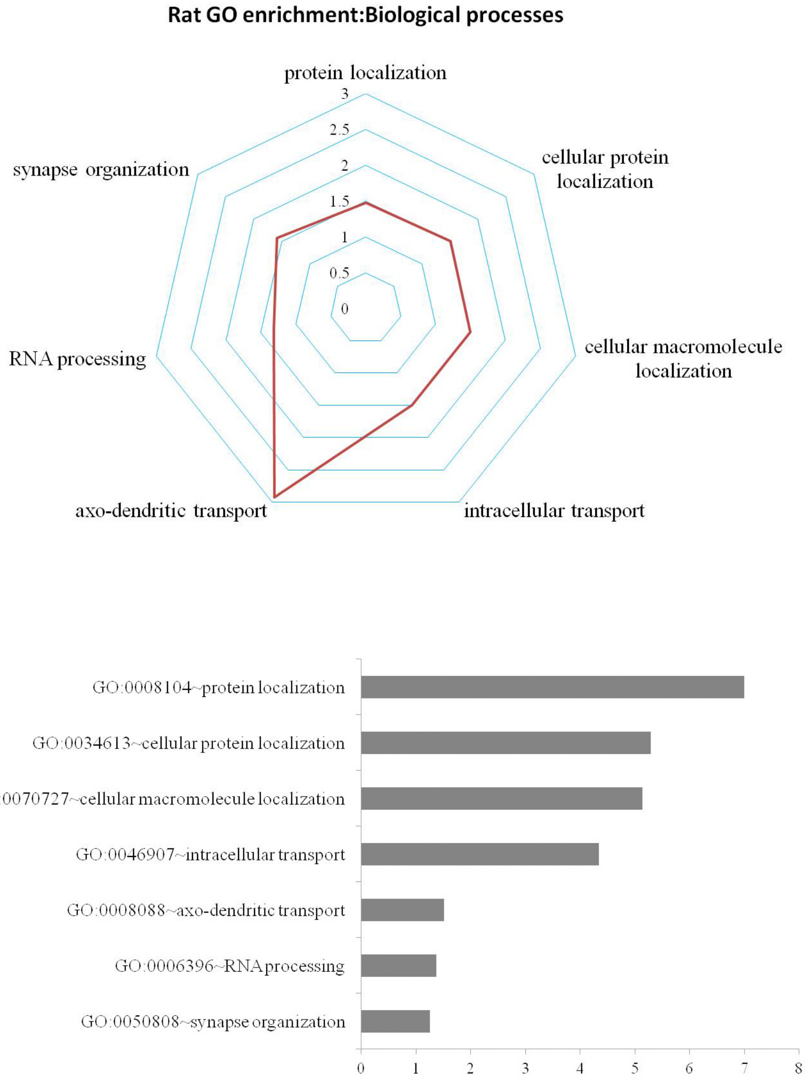

B

Chromatin-related

Histone H2A
BRD4 (acetylated Histone)
CHD7 (methylated H3)
CHD1 (H3K4me2 and H3K4me3)
CHD2 (H3.3)
PARP1 (H3K4me3)
WAC (H2B ubiquitination)
ATRX (SWI/SNF complex)
ARID1B (SWI/SNF complex)
TOP1

Transcriptional factor

GTF2A1
GATA6
POU3F3
POU4F1
YY1
NFX1
ZFP37
SOX21
ZNF652
CDX2
CREB5
TBX1
SIN3A
FBRSL1 (PRC1 complex)
CBX4 (PRC1 complex)
AUTS2 (PRC1 complex)
PHF6
ATN1
PFN1

Splicing factor

HNRNPA3	hnRNP particles
HNRNPL	hnRNP particles
HNRNPA2B1	hnRNP particles
HNRNPA1	hnRNP particles
SF3A2	U2 snRNP complex
SF3B4	U2 snRNP complex
DDX42	U2 snRNP complex
DHX15	U11/U12 snRNPs
SFPQ	U5.4/6 snRNP complexes
NONO	U5.4/6 snRNP complexes
PTBP1	Interacts with RAVER1 and SFPQ
TIA1	alternative pre-RNA splicing
SF1	Binds U2AF2. Interacts with U1 snRNA. Binds EWSR1, FUS and TAF15
CPSF7	pre-mRNA 3'-processing
WDR33	pre-mRNA 3'-processing
PABPC1	PolyA-related
ATXN2	Interacts with PABPC1
ATXN2L	
SFSWAP	
SAFB2	
RBM33	
RBM27	
RBM15B	

Supplementary Table 4. Overlap between genes containing MeCP2-regulated exons and different gene lists

Gene list	Gene number	overlap	*Odd ratio*	*P-value*
Postsynaptic proteome	1080 (974)	163	2.87	0
NMDAR complex	186(172)	36	3.31	1.13e-8
mGluR5 complex	52(49)	16	5.11	1.195e-6
AMPAR complex	9(6)	1	2.58	0.355
synaptome	152(144)	35	3.85	6.40e-10
Presynaptic proteome	393(304)	54	2.84	5.32e-10
SFARI AUTISM	616(513)	64	1.99	2.21e-6
